# Turbulence Model Sensitivity and Scour Gap Effect of Unsteady Flow around Pipe: A CFD Study

**DOI:** 10.1155/2014/412136

**Published:** 2014-07-20

**Authors:** Abbod Ali, R. K. Sharma, P. Ganesan, Shatirah Akib

**Affiliations:** ^1^Department of Mechanical Engineering, Faculty of Engineering, University of Malaya, 50603 Kuala Lumpur, Malaysia; ^2^Department of Civil Engineering, Faculty of Engineering, University of Malaya, 50603 Kuala Lumpur, Malaysia

## Abstract

A numerical investigation of incompressible and transient flow around circular pipe has been carried out at different five gap phases. Flow equations such as Navier-Stokes and continuity equations have been solved using finite volume method. Unsteady horizontal velocity and kinetic energy square root profiles are plotted using different turbulence models and their sensitivity is checked against published experimental results. Flow parameters such as horizontal velocity under pipe, pressure coefficient, wall shear stress, drag coefficient, and lift coefficient are studied and presented graphically to investigate the flow behavior around an immovable pipe and scoured bed.

## 1. Introduction 

Scouring is a phenomenon caused by erosion of sediment of sand bed around an obstruction, that is, bridge piers and abutments in a flow field [1–3]. In other words, scouring basically happens due to the movement of the foundation of the bed under the flow filed conditions in which the flow surrounding the obstruction gets accelerated and induces high shear stress over the seabed surface [4]. The sand bed reduced around the obstruction under the flow level is named the scour depth. A scour hole is a pit or void that forms as a sequence of the sand bed sediment removal from the river bed [5].

Prediction of scour around bridge piers and submarine pipelines attract the hydraulic and ocean engineers. Cylindrical bridge piers are the most commonly used structures in coastal, offshore, and river engineering. Local scouring around the bridge piers is considered to be one of the most common causes of bridge failure [6, 7]. The local scour around river hydraulic structures is a disaster mitigation of the engineering structure [8]. It leaves them in unsafe conditions requiring maintenance and occasionally results in loss of life. Damage of hydraulic structure because of local scouring is a global concern and it has been studied by many researchers experimentally and numerically for several decades. For bridge engineering practice, accurate prediction of local scour, such as the maximum depth of scour around the bridge piers, is critical for bridge design, maintenance, and evaluation. Scour surrounding obstructions, that is, bridge piers and abutments, are considered as a common reason causes bridge failures compared to other causes in the history [9, 10]. The flow in the horizontal channels e.g. river obstructed by the vertical column gets separated, and when viewed from the top, looks like the shoe of a horse. Because of the occurrence of such shape, it is known as horseshoe vortex. This separated layer rolls up along the bridge piers to form a vortex which is known as horseshoe vortex because of its shape.

Mao [11] studied the interaction between a pipeline and erodible bed. Author observed the scour around horizontal cylinders in steady current and wave conditions, as well as with different Reynolds numbers (Re), Shields parameters, and pipeline gaps. These experiments investigated scour features such as shape and size of the scour hole and the time scale of scour formation. Later this work was further investigated by Jensen et al. [12]. They investigated experimentally the flow around a pipeline placed initially on a flat, erodible bed at five characteristics stages of the progressive process in currents. The results showed that as the scour develops with time and space, the mean flow field and turbulence around and the forces on a pipeline undergo considerable changes.

Experimental observation [13] revealed that the horseshoe vortices play the basic role in the scour around the bridge piers. The first scouring takes place in the wake of the cylinder. The primary wake vortices and the accelerated side flow are the main cause of this scouring. A CFD study to predict the local scour hole around the cylindrical pier was carried out by [14]. They found that the CFD methods with the powerful flow visualization show the ability of flow representation during local scouring. Authors concluded that the model used is sufficient to predict the complicated flow field around piers that mounted on sandy bed. In a similar numerical study [15], authors used Eulerian two-phase model coupled with Euler-Euler governing equations for fluid and solid phases. Investigations into the mechanism of scour reveal that three sediment transport modes (bed-load, suspended-load, and laminated-bed) are associated with the scour development.

Roy and Matin [16] experimentally investigated the behavior of scour at floodplain and main channel using three bed materials with three discharges and four length-width ratios. They found that scour behavior at flood plain and main channel for different bed materials, discharges, structure shapes, and length-width ratio is nearly same. They also found that the flood plain is lower in main channel than the flow velocity and as a results, flow velocity is responsible for deeper scour in main channel. The work reported in [17] is a numerical study for simulating a flow field around a circular pier on sandy bed. Reynolds-averaged Navier-Stokes equations (RANS) using standard *k*-*ε* model and space averaged Navier-Stokes equations using large eddy simulation (LES) with the standard Smagorinsky subgrid model were employed to simulate the flow field. It was concluded that RANS simulation is an accurate and sufficient model for scouring in engineering applications and requires lesser grids for simulation. There has been substantial amount of work using finite volume method (FVM) for RANS equations in the various flow condition. del Coz Díaz et al. [18] and Nieto et al. [19] in their work used air as the flowing medium and investigate the performance of snow fence and self-weighted metallic roof using FVM.

Kazeminezhad et al. [20] investigated numerically the force components and vortex shedding frequency of a pipe exposed to a steady current in terms of the drag coefficient, lift coefficient, and Strouhal number. It was concluded that the mean force coefficients and the root-mean-square (RMS) lift coefficient are strongly affected by the gap to diameter ratio while the Strouhal number is slightly affected by the gap ratio. Later in [21] they numerically investigated wave-induced tunnel scour beneath marine pipelines. Investigations revealed that the tremendous sediment transport takes place during the tunnel scour stage and the high turbulence intensity.

This study aims to investigate the effect of turbulence models on the flow field behavior at different five scouring phases and study the effect of scouring on flow parameters such as velocity under pipe, pressure coefficient around the pipe, and wall shear stress on the scour's bed.

## 2. Methodology

### 2.1. Geometrical Structure and Boundary Conditions

**Figure 1:**
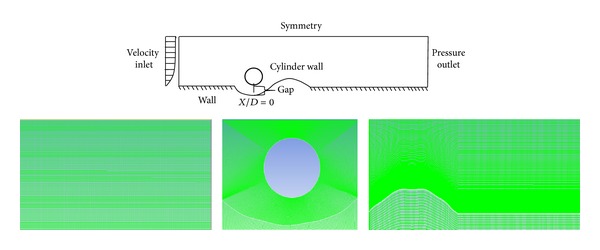
Geometrical model of computational domain and boundary conditions.

Figure 1 shows the schematic of the two-dimensional (2D) geometrical domain used in the present study along with the corresponding boundary conditions. A logarithmic velocity profile as presented in Figure 3 is created using user-defined functions (UDF) in Fluent based on the following formulation [22]:

(1) $$\begin{array}{c} u_{\infty }=\frac{u_{*}}{K}\ln\frac{y}{y_{0}}. \end{array}$$

The velocity profile is applied at the inlet. The profile from our CFD model is compared with that from experimental study of Dudley [23] for consistency; see Figure 3. Zero pressure outlet boundary condition is applied at the flow exit. The water surface (top wall) is set as a symmetry boundary condition. No-slip boundary condition is applied on the pipe surface and the scour bed. Gravity acts in the negative *y*-direction.

### 2.2. Governing Equations

The continuity and the momentum equations for the present case are as given below.

Continuity equation is as follows:

(2) $$\begin{array}{c} \frac{\partial u}{\partial x}+\frac{\partial v}{\partial y}=0. \end{array}$$

*X*-component of the momentum equation is as follows:

(3) $$\begin{array}{c} \rho \left(u\frac{\partial u}{\partial x}+v\frac{\partial u}{\partial y}\right)=-\frac{\partial \rho }{\partial x}+\mu \left(\frac{\partial^{2}u}{\partial x^{2}}+\frac{\partial^{2}u}{\partial y^{2}}\right). \end{array}$$

*Y*-component of the momentum equation is as follows:

(4) $$\begin{array}{c} \rho \left(u\frac{\partial v}{\partial x}+v\frac{\partial v}{\partial y}\right)=-\frac{\partial \rho }{\partial y}+\rho g+\mu \left(\frac{\partial^{2}v}{\partial x^{2}}+\frac{\partial^{2}v}{\partial y^{2}}\right). \end{array}$$

### 2.3. Turbulence Modeling

Turbulence models of two-equation *k*-*ε*, two-equation *k*-*ω* models, and five-equation Reynolds stress models are used in the present investigation and their results are compared with experimental data from literature.

#### 2.3.1. *k*-*ε* Models

Two-equation *k*-*ε* models, turbulent kinetic energy *k* and turbulent dissipation *ε*, are the simplest and the most widely used models among all turbulence models that aim to study the effect of turbulence in the flow. Two-equation model signifies that it includes two extra transport equations to represent the turbulence properties of the flow. There are three different models that are derived from *k*-*ε* model, standard *k*-*ε* model, realizable *k*-*ε* model, and renormalization group method (RNG). Despite having the two common equations, these turbulence models use the different ways to calculate the principle form of the eddy viscosity equation.

The variants *k*-*ε* models approximate the eddy viscosity as

(5) $$\begin{array}{c} \mu_{t}=\frac{\rho C_{u}k^{2}}{\varepsilon }. \end{array}$$

The turbulent kinetic energy (*k*) and its dissipation rate (*ε*) for the standard *k*-*ε* model are calculated from

(6) ∂∂t(ρk)+∂∂xi(ρkui)=∂∂xj[(μ+μtσk)∂k∂xj] +Gk+Gb−ρε+Sk,∂∂t(ρε)+∂∂xi(ρεuj)=∂∂xj[(μ+μtσε)∂ε∂xj] +C1εεk(Gk+C3εGb) −C2εε2k+Sε.

The model constants are *C*_1*ε*_ = 1.44, *C*_2*ε*_ = 1.92, *C*_3*ε*_ = −0.33, *C*_*μ*_ = 0.09, *σ*_*k*_ = 1.0, and *σ*_*ε*_ = 1.3.

The modeled transport equations for (*k*) and (*ε*) in the RNG *k*-*ε* model are

(7) ∂∂t(ρk)+∂∂xi(ρkui)=∂∂xj(αkueff∂k∂xj) +Gk+Gb−ρε+Sk,∂∂t(ρε)+∂∂xj(ρεui)=∂∂xj(αεueff∂ε∂xj) +C1εεk(Gk+C3εGb) −C2ερε2k−Rε+Sε.

The model constants are *C*_1*ε*_ = 1.42, *C*_2*ε*_ = 1.68, and *C*_*μ*_ = 0.084.

The modeled transport equations for (*k*) and (*ε*) in the realizable *k*-*ε* model are

(8) ∂∂t(ρk)+∂∂xj(ρkuj)=∂∂xj[(u+utσk)∂k∂xj] +Gk+Gb−ρε+Sk,∂∂t(ρε)+∂∂xj(ρεuj)=∂∂xj[(u+utσk)∂ε∂xj] +ρC1εSε+ρC2ε2k+νε +C1εεkC3εGb+Sε.

The model constants *C*_2_, *σ*_*k*_, and *σ*_*ε*_ have been established to ensure that the model performs well for certain flows. The model constants are

(9) $$\begin{array}{c} C_{1\varepsilon }=1.44,\;C_{2}=1.9,\;\sigma_{k}=1.0,\;\sigma_{\varepsilon }=1.2. \end{array}$$

#### 2.3.2. *k*-*ω* Models

*k*-*ω* models, also known as two-equation models, have the same definition for *k* as in *k*-*ε* model. However, it differs in the selection of second variable (*ω*). This model is broadly categorized into two types, the standard *k*-*ω* model and the shear stress transport (SST) model.

The turbulent viscosity, *μ*_*t*_, for the *k*-*ω* model, is computed by combining (*k*) and (*ω*) as follows:

(10) $$\begin{array}{c} \mu_{t}=\alpha^{*}\frac{\rho k}{\omega }. \end{array}$$

The modeled transport equations for (*k*) and (*ω*) in the standard *k*-*ω* model are

(11) $$\begin{array}{c} \frac{\partial }{\partial t}\left(\rho k\right)+\frac{\partial }{\partial x_{i}}\left(\rho ku_{i}\right)=\frac{\partial }{\partial x_{j}}\left(\Gamma_{k}\frac{\partial k}{\partial x_{j}}\right)+G_{k}+S_{k},\\ \frac{\partial }{\partial t}\left(\rho \omega \right)+\frac{\partial }{\partial x_{i}}\left(\rho \omega u_{i}\right)=\frac{\partial }{\partial x_{j}}\left(\Gamma_{\omega }\frac{\partial \omega }{\partial x_{j}}\right)+G_{\omega }+S_{\omega }. \end{array}$$

The modeled transport equations for (*k*) and (*ω*) in the SST *k*-*ω* model are

(12) $$\begin{array}{c} \frac{\partial }{\partial t}\left(\rho k\right)+\frac{\partial }{\partial x_{j}}\left(\rho ku_{i}\right)=\frac{\partial }{\partial x_{j}}\left(\Gamma_{k}\frac{\partial k}{\partial x_{j}}\right)+G_{k}+S_{k},\\ \frac{\partial }{\partial t}\left(\rho \omega \right)+\frac{\partial }{\partial x_{j}}\left(\rho \omega u_{j}\right)=\frac{\partial }{\partial x_{j}}\left(\Gamma_{\omega }\frac{\partial \omega }{\partial x_{j}}\right)+G_{\omega }+D_{\omega }+S_{\omega }. \end{array}$$

#### 2.3.3. Reynolds Stress Model (RSM)

Unlike the previous two turbulence models, Reynolds stress model (RSM) abandons the calculation of eddy viscosity and solves the transport equation for Reynolds stresses. It provides four additional equations for 2D flow and seven for 3D flow. Having these additional equations, RSM takes comparatively more effort and time to simulate the flow.

The exact transport equation for the Reynolds stresses, $\left(\rho \bar{u_{i}^{\prime }u_{j}^{\prime }}\right)$, may be written as follows:

(13) ∂∂t(ρui′uj′¯)+∂∂xk(ρukui′uj′¯) =−∂∂xk[ρui′uj′uk′¯+ρ(δkjui′+δikuj′)¯]  +∂∂xk[μ∂∂xk(ui′uj′¯)]−ρ(ui′uk′¯∂uj∂xk+uj′uk′¯∂ui∂xk)  −ρβ(giuj′θ¯+gjui′θ¯)+ρ′(∂ui′∂xj+∂uj′∂xi)  −2μ∂ui′∂xk∂uj′∂xk¯−2ρΩk(uj′um′¯εikm+ui′um′¯εjkm)  −2ρΩk(uj′um′¯εjkm+ui′um′¯εjkm).

### 2.4. Numerical Methods

The commercial CFD software FLUENT 14.0 [24] which is based on finite volume method (FVM) is used to solve the Reynolds-averaged Navier-Stokes (RANS) equations for the incompressible flow. The transport governing equations are discretized using the second order upwind spatial discretization method. The pressure-implicit with splitting of operators (PISO) scheme was used for the coupling of the pressure and the velocity fields. The underrelaxation factor of all the components, such as velocity components and pressure correction, is kept at 0.3. The scaled residuals of 1 × 10^−6^ are set as the convergence criteria for the continuity and momentum equations. Transient model based on implicit scheme with a time step was used in the current numerical investigation. The typical wall treatment function *y* ^+^ (=*yU*_*τ*_/*ν*) value of the first node in all turbulence models near the bed profile is less than 1.

### 2.5. Simulation Cases

A total of 11 cases were simulated in the present study; see Table 1. In the first section of this study, the effect of different turbulence models such as *k*-*ε* models (standard, RNG, and realizable), *k*-*ω* models (SST, standard), and Reynolds stress model (RSM) on horizontal velocity and kinetic energy square root has been investigated. For studying this effect, we have adopted the domain suggested by Jensen et al. [12] to validate the results of Mao [11]. This domain is of 0.5 m length and 0.1 m height with pipe diameter of 0.03 m. Turbulence models were tested for scour gap at time 0 min, 1 min, 6 min, 30 min, and 300 min and four positions (*X*/*D* = −3.0, 1.0, 1.5, and 2.0) in-front and behind the pipe. Qualitatively the results of a particular turbulence model were the same for all scour gaps; so, the results of cases 1–6 for scour gap at time 30 min are presented. Consequently, the turbulence model that reproduces a similar result as of experimental investigation of Jensen et al. is chosen for all further simulation cases in the current study.

In the second part of this paper, a parametric study has been carried out by using five bed profiles suggested by Mao [11] at 10 min, 30 min, 100 min, 200 min, and 300 min. Five simulation cases (7–11) were run to obtain the horizontal velocity profile under the pipe, pressure coefficient *C*_*p*_, wall shear stress *τ*_*x*_, drag coefficient *C*_*d*_, and lift coefficient *C*_*l*_ around the pipe with different gaps at position *X*/*D* = 0 using standard *k*-*ε* turbulence model.

### 2.6. Mesh Independency and Time Step Test

**Figure 2:**
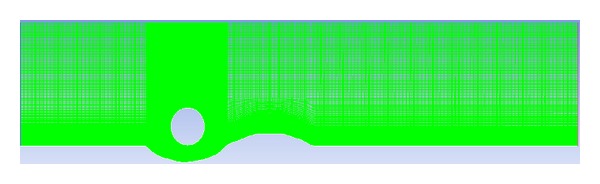
The grid for the model calculation.

**Figure 3:**
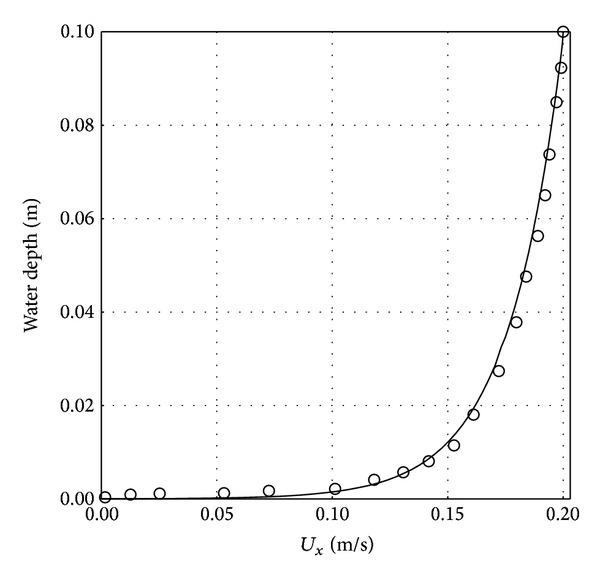
Comparison of the horizontal logarithmic velocity inlet (*U*_0_) in the total water depth between present numerical investigation and experimental work of Dudley [23].

**Figure 4:**
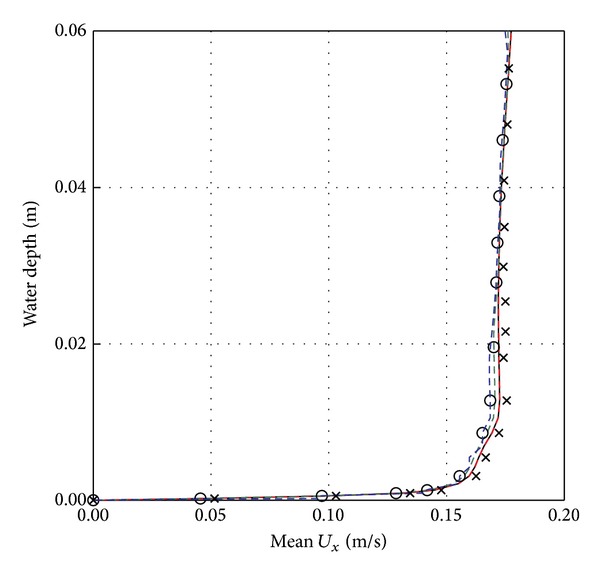
Mesh independence test for mean *U*_*x*_  (m/s) at location *X*/*D* = −3.0 for different types of grid cell numbers, Red Jensen, Blue 40000, ○ 82500, Green 130225, Black 223404, × 315623.

The domain is meshed using a structured grid; see Figure 2. Mesh independency is carried out using five different types of grids having cell number of 40000, 82500, 130225, 223404, and 315623. A finer grid is used near the bed. The mean *U*_*x*_ velocity was calculated for all selected mesh sizes and plotted against the water depth as shown in Figure 4. The domain with 223,404 cells is selected throughout this study, because it shows reasonable accuracy and the lowest deviations of the velocity profile and turbulent kinetic energy (TKE) for turbulence models compared to Jensen laboratory data [12]. Four different time step sizes (Δ*t* = 0.0002, 0.002, 0.02, and 0.2) were tested and 0.002 is used throughout this current numerical study as there were no deviations observed below this value of time step. The number of iterations for this study is kept between 3000 and 3500.

## 3. Results and Discussion

Current numerical study has been carried out in two major sections. First section deals with the effect of various turbulence models on horizontal velocity profile and kinetic energy square root while the second one deals with the effect of scour gap on velocity profile under the pipe, wall shear stress, and others. A large number of simulations have been run to study the effect of turbulence models on unsteady horizontal velocity profile and turbulent kinetic energy square root, and the results are discussed in Section 3.1. The preferred turbulence model identified using above study is used for further investigation of effect of scour gap on velocity profile under the pipe, *C*_*p*_, wall shear stress, *C*_*d*_, and *C*_*l*_ which is discussed in Section 3.2.

### 3.1. Effect of Different Turbulence Models

**Figure 5:**
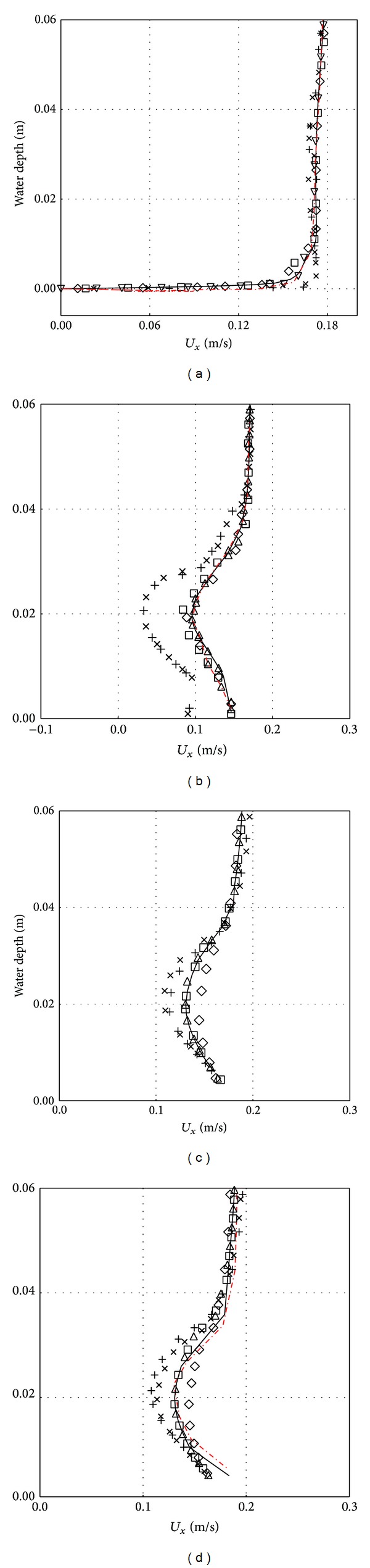
Unsteady horizontal velocity (*U*_*x*_) at different positions, (a) *X*/*D* = −3.0, (b) *X*/*D* = 1.0, (c) *X*/*D* = 1.5, and (d) *X*/*D* = 2.0 with different turbulent models — standard *k*-*ε*, ⋄ RNG *k*-*ε*, △ realizable *k*-*ε*, × standard *k*-*ω*, + SST *k*-*ω*, and □ Reynolds stress model (RSM) Red Jensen.

**Figure 6:**
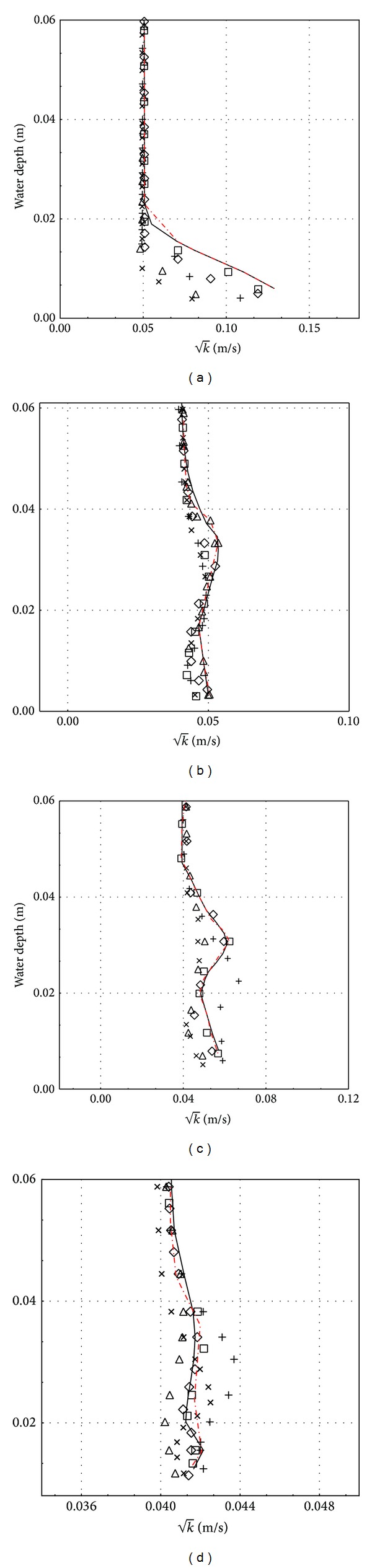
Turbulent kinetic energy square root ($\sqrt{k}$) at different locations, (a) *X*/*D* = −3.0, (b) *X*/*D* = 1.0, (c) *X*/*D* = 1.5, and (d) *X*/*D* = 2.0, — standard *k*-*ε*, ⋄ RNG *k*-*ε*, △ realizable *k*-*ε*, × standard *k*-*ω*, + SST *k*-*ω*, and □ Reynolds stress model (RSM), Red Jensen.

Cases 1–30 are used to predict the horizontal velocity profile (*U*_*x*_) and the turbulent kinetic energy (TKE) at some axial length (i.e., *X*/*D* = −3.0, 1.0, 1.5, and 2.0) using different type of turbulence models and the results are presented in Figures 5 and 6, respectively. For comparisons, the experimental results reported in Chang [2] are also presented in the figure. Note that the location of the axial position covers the front and rear part of the pipe (or obstruction). Referring to Figures 5(a) to 5(d), which is presented at *X*/*D* = −3.0, 1.0, 1.5, and 2.0, respectively, the standard *k*-*ɛ* turbulence model prediction is much closer to the experimental data at various water depths; some of them overlap each other. Nearly the same can be said for the realizable *k*-*ɛ* model and RSM model but some deviation seen at some of water depths, for example, at water depths below 0.01 m and those between 0.03 and 0.04 m at *X*/*D* = 2.0 (Figure 5(d)) for the both models. RNG *k*-*ɛ* model prediction is quite accurate for *X*/*D* = −3.0 and 1.0 (Figures 5(a) and 5(b)), but this is not the case for *X*/*D* = 1.5 and 2.0 (Figures 5(c) and 5(d)); overprediction of the velocity is seen at water depths between 0.01 and 0.03 m. Relatively, standard *k*-*ω* and SST *k*-*ω* models are the most inaccurate among the turbulence models studied. Significant under prediction of the velocity is seen at water depths from 0 to 0.04 m at *X*/*D* = 1.0, 1.5, and 2.0. Referring to Figure 6, the TKE is well predicted by standard *k*-*ɛ* turbulence with some deviation at *X*/*D* = 2.0 (Figure 6(d)) in comparison to other turbulence models. RNG *k*-*ɛ* model and RSM model also reasonably predict TKE especially for *X*/*D* = 1.5 and 2.0 and realizable *k*-*ɛ* model has a good prediction only at *X*/*D* = 1.0. On overall, realizable *k*-*ɛ* model, standard *k*-*ω*, and SST *k*-*ω* models can be regarded as the least accurate among the turbulence models studied in predicting the turbulence kinetic energy.

Among all turbulence models, standard *k*-*ε* model gives better predictions for the velocity profile when compared with that of experimental data of Jensen et al. Standard *k*-*ε* model is widely acceptable for scour process modelling because of its better accuracy and reasonable computation time and does not require high computational facilities. However, the *k*-*ω* models show a discrepancy with the experimental data and the velocity profile deviates largely near the wake region (highly turbulent region) and less in the farther region in the downstream profile. This deviation is more significant at the bottom (near the wall bed) and around the pipeline and reduces towards the water surface. This deviation may be because *k*-*ω* model produces slightly large turbulence in the weak region which is less encountered with the variants *k*-*ɛ* models and the Reynolds stress model (RSM). It may also be because of the absence of the wall treatment function in *k*-*ω* models, and they are also very sensitive to the inlet boundary conditions flow.

### 3.2. Effect of Scouring Depth

**Figure 7:**
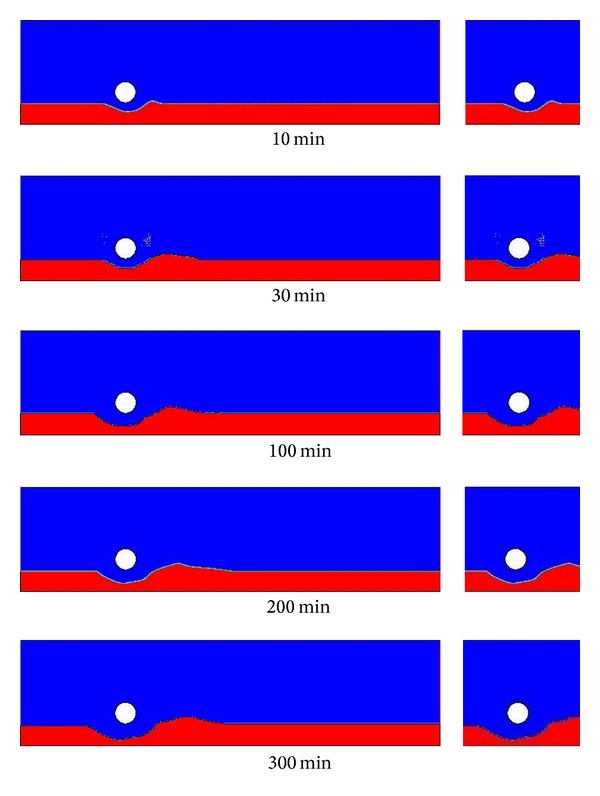
Bed profiles during the development of scouring.

**Figure 8:**
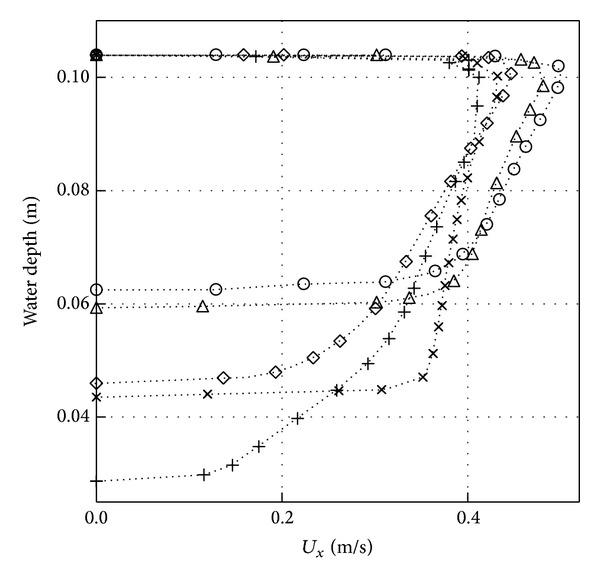
Horizontal velocity profile (*U*_*x*_) in the gap at (*X*/*D* = 0) and at different scour gaps, ○ 10 min, △ 30 min, ⋄ 100 min, × 200 min, and + 300 min.

Figure 8 shows the horizontal velocity profile under the pipe (obstruction) at *X*/*D* = 0 for different types of scoured bed profiles as presented in Figure 7. A higher horizontal velocity can be expected for the scoured bed profiles of 10 min and 30 min due to a narrow gap under the pipe to the surface of the bed. The opposite can be said for the scoured bed profiles of 100, 200, and 300 min. A reduction of around 18% of horizontal velocity is observed from time phase from 10 min to 300 min.

**Figure 9:**
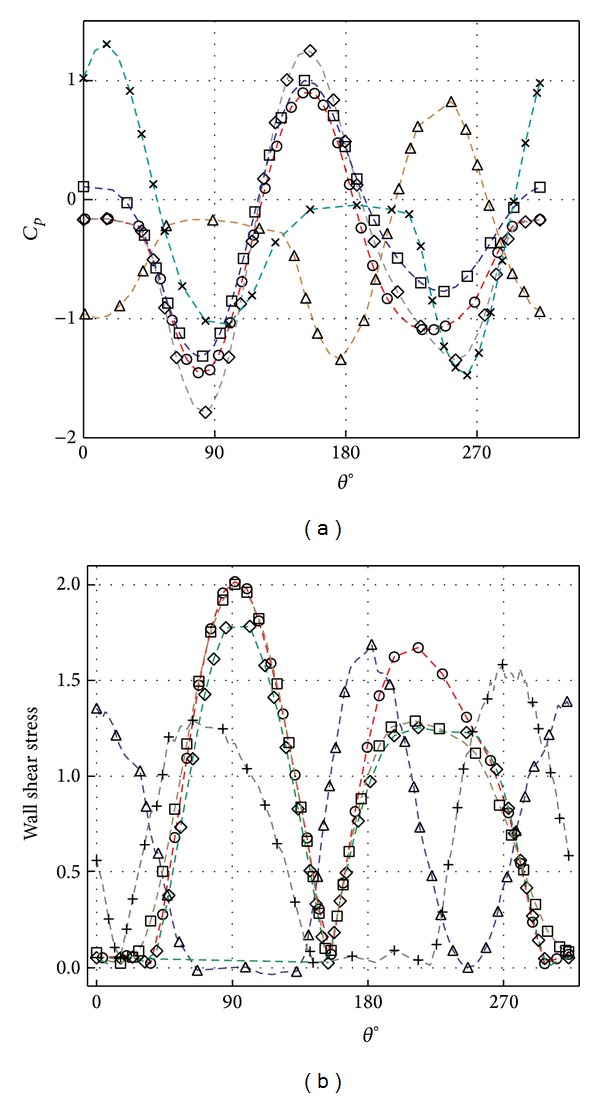
Effect on (a) pressure coefficient (*C*_*p*_) and (b) wall shear stress at cylinder surface of different scour gaps, ○ 10 min, △ 30 min, ⋄ 100 min, □ 200 min, and × 300 min.

Figure 9 shows the prediction of the pressure coefficient and wall shear stress variation around the pipe at five different circumferential points (*θ*). The variation of the pressure coefficient with gap depth at different circumferential positions is shown in Figure 9(a). The angle *θ* indicates the position of the pressure coefficient around the pipe surface from 0° the point nearest to the wall to 360° in the clockwise direction. The stagnation and separation points which can be referred as maximum and minimum peak are clearly delineated in the figure. The positive value of the pressure coefficient indicates that the pressure rises and the water level increases at that location, whereas the negative value indicates that the pressure drops and the water level decreases.

Figure 9(b) shows the variation of the wall shear stress with gap depth. The wall shear stress forms as a sequence of approaching the flow on the pipe parallel to bed surface and it experiences the maximum value *τ*_max⁡_ at the flow separation point which causes scouring to begin. At the early stage, *τ*_max⁡_ occurs near the bottom in each side of the pipe forming shedding vortex that creates a space on both sides of the pipe which is soon occupied with water and leads the scour formation. Further, the maximum shear stress moves downward approaching the bed and moves the sand particles in the flow direction. It is observed that the higher the shear stress, the deeper the scour hole below the pipe.

**Figure 10:**
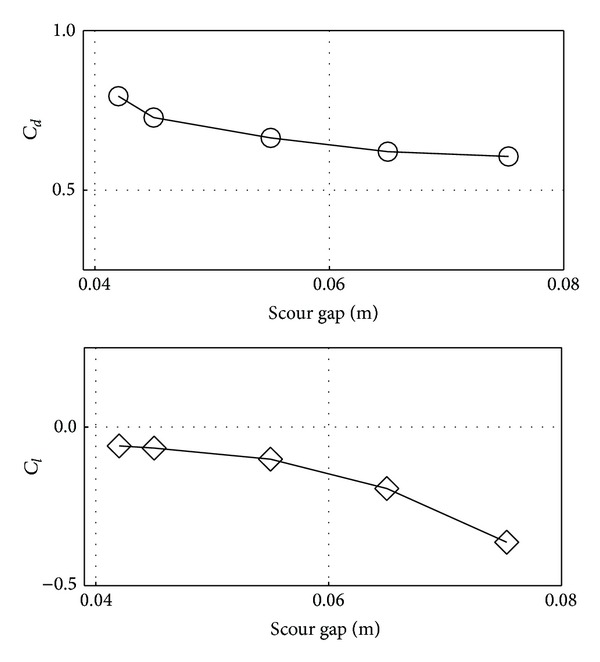
Effect on (a) drag coefficient (*C*_*d*_) and lift coefficient (*C*_*l*_) at cylinder surface of different time phases.

When the flow approaches the pipe, it imparts drag and lift forces on the pipe. In the current numerical study, variations in these forces are observed in terms of drag and lift coefficients for five different time phases and presented in Figure 10. Shear stress causes the bed erosion and as soon as it comes into action, the pipe experiences negative lift coefficient (*C*_*l*_) and it decreases with time and consequently increases the scour gap. The negative lift can be attributed to the suction below and behind the pipe caused by the scour gap. For velocity, negative lift can be explained by the position of the stagnation point pipe and the angle of attack of the approaching flow. As a result of lift coefficient elimination, the drag coefficient is reduced with time as well. A reduction of 23.7% in *C*_*d*_ and 51.3% in *C*_*l*_ was observed between the time phases of 10 min and 300 min.

## 4. Conclusions

Two-dimensional (2D) CFD analyses were carried to investigate fluid flow over an obstruction under different type of bed profiles using a number of turbulence models. Unsteady horizontal velocity profile and the kinetic energy square root at few axial directions are investigated. The effect of scour depth on the velocity distribution under the pipe, the wall shear on the bed and the pressure coefficient, the drag coefficient, and the lift coefficient of the obstruction body were numerically investigated. The conclusions of the current study are as follows.

- The standard *k*-*ε* model is able to predict accurate results in comparison to other turbulence models when compared to experimental data for unsteady horizontal velocity and turbulent kinetic energy square root.
- From the velocity behavior under the pipe, results show that the maximum velocity at each phase decreases with increasing time till the scour reaches its equilibrium depth and a reduction of around 18% of horizontal velocity is observed from time phase from 10 min to 300 min.
- The drag and lift coefficients decrease as the gap under the pipe increases. A reduction of 23.7% in *C*_*d*_ and 51.3% in *C*_*l*_ was observed between the time phases of 10 min and 300 min.

## Figures and Tables

**Table 1:** Simulation cases.

Sim.∗	Domain	Cases	Turbulence models	Positions (*X*/*D*)	Remarks
1	(1, 2, 3, 4, and 5) (0, 1, 6, 30, and 300 min)	1–6	Standard *k*-*ε* RNG *k*-*ε* Realizable *k*-*ε* Standard *k*-*ω* SST *k*-*ω* RSM	3.0, 1.0, 1.5, and 2.0	To identify the preferred turbulence model

2	(1, 2, 3, 4, and 5) (10, 30, 100, 200, and 300 min)	7–11	Standard *k*-*ε*	0	To evaluate velocity profile under the pipe, *C*_*p*_, wall shear stress, *C*_*d*_, and *C*_*l*_

*Simulation.

## Nomenclature

CFD: Computational fluid dynamic
*C*_1*ε*_, *C*_2*ε*_: Model constants
*C*_*d*_: Drag coefficient
*C*_*l*_: Lift coefficient
*C*_*s*_: Roughness constant (m)
*C*_*p*_: Pressure coefficient *C*_*p*_ = (*P* − *P*_0_)/(0.5*u*_0_^2^)
*D*: Cylinder diameter (m)
FVM: Finite volume method
*g*: Gravitational acceleration (m/s²)
*G*: Gap between maximum scoured bed and cylinder (m)
*G*_*b*_: The generation of turbulence kinetic energy due to buoyancy
*G*_*k*_: The generation of turbulence kinetic energy due to the mean velocity gradients
*K*: von Karman constant, (= 0.41)
*k*: Turbulent kinetic energy (TKE) (m^2^s^2^)
$\sqrt{k}$: Turbulent kinetic energy square root (m/s)
PISO: Pressure-implicit with splitting of operators
*P*: Dynamic pressure (Reynolds average) (Nm²)
*P*_0_: Reference pressure (Nm²)
RANS: Reynolds-averaged Navier-Stokes equations
Re: Reynolds numbers
RNG: Renormalization group method
RSM: Reynolds stress model
SST: Shear stress transport
UDF: User-defined functions
*U*_0_: *X*-direction velocity-inlet component (m/s)
*U*_*∞*_: Approach velocity (m/s)
*u*_∗_: Friction (or shear) velocity, *u*_∗_ = (*τ*/*ρ*)^1/2^ (m/s)
$\bar{u_{i}^{\prime }u_{j}^{\prime }}$: Turbulent momentum flux or Reynolds stress (*i*, *j* component)
*u*_*i*_: Flow velocity in *i*-direction (Reynolds average) (m/s)
*u*_*i*_′: Flow velocity in *i*-direction (fluctuating part) (m/s)
*u*_*j*_: Flow velocity in *j*-direction (Reynolds average) (m/s)
*u*_*j*_′: Flow velocity in *j*-direction (fluctuating part) (m/s)
*y*: Water (or flow) depth (m)
*y*_0_: Roughness height (m)
*y*^+^: Wall distance estimation (m)
*ε*: Rate of dissipation of turbulent kinetic energy (*k*) (m²/s³)
*ω*: Specific dissipation (1/s)
*τ*_*w*_: Shear stress
Δ*t*: Time step (s)
*θ*: The angle indicates the position around the cylinder's surface (°)
*v*: Velocity (m/s)
Γ_*k*_ and Γ_*ω*_: Represent the effective diffusive of *k* and *ω*
*Y*_*k*_ and *Y*_*ω*_: Represent the dissipation of *k* and *ω* to turbulence
*ρ*: Water density (m/s^3^)
*μ*_*t*_: The turbulent (or eddy) viscosity (Ns/m²)
*μ*: Dynamic viscosity (Ns/m²)
$\left(\rho \bar{u_{i}^{\prime }u_{j}^{\prime }}\right)$: Reynolds stresses
*G*_*k*_, *S*_*k*_: Source items
*G*_*ω*_ + *D*_*ω*_ + *S*_*ω*_: Source items.
